# A randomized prospective cross over study on the effects of medium cut-off membranes on T cellular and serologic immune phenotypes in hemodialysis

**DOI:** 10.1038/s41598-022-20818-z

**Published:** 2022-09-30

**Authors:** Georg Lorenz, Yuli Shen, Renate Ilona Hausinger, Caroline Scheid, Marie Eckermann, Sophia Hornung, Joana Cardoso, Maciej Lech, Andrea Ribeiro, Bernhard Haller, Christopher Holzmann-Littig, Dominik Steubl, Matthias C. Braunisch, Roman Günthner, Andreas Poschenrieder, Britt Freitag, Mario Weber, Peter Luppa, Uwe Heemann, Christoph Schmaderer

**Affiliations:** 1 Department of Nephrology, School of Medicine, Klinikum rechts der Isar, Technical University of Munich, Ismaninger Str. 22, 81675 Munich, Germany; 2 Department of Nephrology, LMU Klinikum, Ludwig-Maximilian University, Munich, Germany; 3 Institute for AI and Informatics in Medicine, Klinikum rechts der Isar, Technical University Munich, Munich, Germany; 4 Department of Clinical Chemistry, Klinikum rechts der Isar, Technical University Munich, Munich, Germany; 5 MVZ KfH-Gesundheitszentrum Emmering/Dachau, Dachau, Germany; 6 Nephrology Department of The Second Affiliated Hospital, School of Medicine, The Chinese University of Hong Kong, Shenzhen, 518172 Guangdong People’s Republic of China; 7 Longgang District People’s Hospital of Shenzhen, Shenzhen, People’s Republic of China

**Keywords:** Inflammation, Chronic inflammation, Haemodialysis, T cells

## Abstract

Extended cut-off filtration by medium cut-off membranes (MCO) has been shown to be safe in maintenance hemodialysis (HD). The notion of using them for the control of chronic low-grade inflammation and positively influencing cellular immune aberrations seems tempting. We conducted an open label, multicenter, randomized, 90 day 2-phase cross over clinical trial (MCO- vs. high flux-HD). 46 patients underwent randomization of which 34 completed the study. Dialysate- or pre- and post-dialysis serum inflammatory mediators were assayed for each study visit. Ex vivo T cell activation was assessed from cryopreserved leucocytes by flow cytometry. Linear mixed models were used to compare treatment modalities, with difference in pre-dialysis serum MCP-1 levels after 3 months as the predefined primary endpoint. Filtration/dialysate concentrations of most mediators, including MCP-1 (mean ± SD: 10.5 ± 5.9 vs. 5.1 ± 3.8 pg/ml, *P* < 0.001) were significantly increased during MCO- versus high flux-HD. However, except for the largest mediator studied, i.e., YKL-40, this did not confer any advantages for single session elimination kinetics (post-HD mean ± SD: 360 ± 334 vs. 564 ± 422 pg/ml, *P* < 0.001). No sustained reduction of any of the studied mediators was found neither. Still, the long-term reduction of CD69+ (*P* = 0.01) and PD1+ (*P* = 0.02) activated CD4+ T cells was striking. Thus, MCO-HD does not induce reduction of a broad range of inflammatory mediators studied here. Long-term reduction over a 3-month period was not possible. Increased single session filtration, as evidenced by increased dialysate concentrations of inflammatory mediators during MCO-HD, might eventually be compensated for by compartment redistribution or increased production during dialysis session. Nevertheless, lasting effects on the T-cell phenotype were seen, which deserves further investigation.

## Introduction

Across Europe, both incidence and prevalence of end stage renal disease have continuously increased. Thereby, hemodialysis (HD) is by far the most used renal replacement therapy^1^. If one leaves aside the influence of the current COVID-19 pandemic, mortality has declined slightly over the last decades, presumably due to better technology and care programs^2,3^. Still, patients on maintenance HD continue to show excess mortality, high burden of metabolic, cardiovascular and infectious disease as well as poor quality of life^4^. Residual uremia, insufficient cytokine or middle molecule clearance, and in vivo blood-membrane interaction give rise to chronic low-grade inflammation, which has been causally linked to excess mortality^5,6^. E.g., IL-6 promotor polymorphisms and consecutively elevated serum IL-6 levels have been associated with cardiovascular (CV) events in chronic kidney disease (CKD)^7^. Further, a plethora of soluble inflammatory mediators including C-reactive protein, Tumor necrosis factor α (TNFα), YKL-40 and atherogenic chemokines, i.e. MCP-1, CCL-5, IP-10 have been associated with adverse events or mortality in HD patients^8–14^.

It seems hardly surprising, that severe disturbances of adaptive cellular immunity have been reported, vaccine responses tend to be poor and breakthrough infections are common in HD populations^15,16^. Despite nearly normal leucocyte counts, HD T-cells demonstrate reduced proliferation responses ex vivo. Further, antigen presenting cell (APC)—T cell interaction is impaired by reduced CD28 co-receptor expression. On the other hand, higher frequencies of CD69+ pre-activated CD4+ and CD8+ cells which are prone to apoptosis were found^17^. More recently, the concept of T cell exhaustion in chronic inflammatory conditions such as tumours and chronic infections has been introduced^18^: If the inflammatory stimulus cannot be eliminated, instead of differentiating into proper memory cells, some T cells take on a fatigued state and lose their ability to mount cytokine responses or proliferate. Furthermore, these cells increasingly express the inhibitory co-receptor programmed cell death protein (PD)-1, which dampens the APC—T cell interaction^18^. Intriguingly, a higher number of PD-1 positive T cells can already be detected in paediatric HD patients^19^.

So, what to do?—In theory, HD permits filtration of inflammatory mediators up to a certain cut off and thus the reduction of chronic inflammatory stimuli. MCO membranes (such as Theranova 400) appear to be particularly suitable for this purpose, as they have a more uniform distribution of a higher pore sizes, which provides a cut off range between the upper range of inflammatory mediators and albumin^20,21^. Safety, improvement or stability of certain quality of life domains and enhanced elimination of λ light chain (45 kDa) versus high flux HD treatment have already been demonstrated for these novel membranes^20,22–24^. Safety concerns with regards to significant albumin drainage due to increased pore size have been ruled out by independent investigators over a 24 weeks period^20,24^.

However, data on long-term elimination of inflammatory mediators remain controversial and it is unclear whether T-cellular immune aberrations can be positively affected^20,25,26^.

Therefore, this randomized prospective cross-over study comparing MCO vs. standard high flux HD was designed to assess single session and long term (3 months) inflammatory serum mediator kinetics. Reduction of Monocyte chemoattractant protein-1 (MCP-1) was the primary endpoint. Impact on peripheral T cell activation was a secondary endpoint.

## Results

### Study participants

The study was conducted between September 2017 and August 2018. A total 46 patients were included and underwent randomization (intention to treat (ITT) population). Of these 34 patients completed the whole study course (per-protocol population (PPP)). By assessing these pre-defined 34 patients, the study provided a power of 80% to detect a difference of 70% of the within subject standard deviation of MCP-1 (see “Methods” section for power calculation). Baseline characteristics of the ITT population are displayed in Table 1. Median patients’ age was 68 (IQR 59, 78) years, 33 (72%) of which were male. Except for a trend towards higher session durations and an enrichment of permanent central venous lines as access type 8 (38%) versus 2 (8%), chi square = 0.03) in the MCO → high flux versus high flux → MCO group, the randomization was balanced. It should be noted that access types did not change in any patient during the study period. Delivered “dialysis dose” (Kt/V) was comparable between the two sequences (Table 1; LMM regression with Kt/V as the dependent variable and “sequence” as main effect: *P* = 0.82). Comparable results were obtained for the PPP (Supplementary Table 2).

**Table 1:** Baseline characteristics of the intention to treat population.

Parameter	Missing data	Overall	MCO → Fx60/80	Fx60/80 → MCO	*P* value
Number of patients	–	46	25	21	–
Age (years)	–	68 [59, 78]	73 [59, 79]	67 [54, 77]	0.29
Sex, male (n%)	–	33 (72%)	19 (76%)	14 (67%)	0.53
BMI (kg/m^2^)	–	26 ± 5	28 ± 5	25 ± 6	0.90
Upper arm circumference (cm)	9	29 ± 5	30 ± 5	29 ± 5	0.25
**Cause of CKD**	–				0.27
Diabetes/hypertension	–	20 (44%)	13 (52%)	7 (33%)	–
Glomerulonephritis		8 (17%)	6 (24%)	2 (10%)	–
Systemic disease		1 (2%)	0 (0%)	1 (5%)	–
Hereditary cause		9 (20%)	4 (16%)	5 (24%)	–
Other		8 (17%)	2 (10%)	6 (29%)	–
CCI adjusted by Liu et al.	–	9 [6, 11]	9 [6, 12]	9 [5, 10]	0.60
Diabetes mellitus (n%)	–	16 (35%)	8 (32%)	8 (38%)	0.76
History MI (n%)	–	13 (28%)	5 (20%)	8 (38%)	0.21
Coronary heart disease (n%)	–	21 (46%)	12 (48%)	9 (43%)	0.77
Atrial fibrillation (n%)	–	18 (39%)	11 (44%)	7 (33%)	0.55
COPD (n%)	–	13 (28%)	8 (32%)	5 (24%)	0.15
Kt/V	3	1.57 ± 0.34	1.54 ± 0.32	1.61 ± 0.37	0.54
HD vintage [months]	–	37 [26, 93]	46 [30, 96]	37 [17, 88]	0.60
Residual renal function [ml], n = 15	–	720 [250, 1090]	760 [325, 1238]	400 [250, 1090]	0.69
Access (catheter)	–	10 (22%)	8 (38%)	2 (8%)	**0.03**
Anticoagulation (citrate/agatra n = 1)	–	7 (15%)	4 (16%)	3 (14%)	0.64
Effective session duration [h]	–	4.3 ± 0.4	4.4 ± 0.5	4.2 ± 0.2	0.51
Ultrafiltration rate [ml/h]		542 ± 207	549 ± 214	533 ± 202	0.88
Blood flow rate [ml/min]	–	236 ± 44	236 ± 45	237 ± 44	0.62
Dialysate flow [ml/min]	–	394 ± 127	399 ± 134	389 ± 122	0.82
Dialysate Na+ [mmol]	–	138 [138, 138]	138 [138, 138]	138 [138, 138]	0.70
Dialysate K+ [mmol]	–	3 [2, 3]	2 [2, 3]	3 [2, 3]	0.08
Dialysate Mg2+ [mmol]	–	1 [0.75, 1]	1 [0.75, 1]	0.75 [0.75, 1]	0.09
Dialysate Ca2+ [mmol]	–	1.25 [1.25, 1.25]	1.25 [1.25, 1.25]	1.25 [1.25, 1.25]	0.37
Dialysate HCO3− [mmol]	–	32 [32]	32 [32]	32 [32]	0.92
Dialysis unit 1 (n%)	–	17 (37%)	7 (28%)	10 (48%)	0.23
Immunosup. (n%)	–	5 (11%)	3 (12%)	2 (10%)	0.39
Statin (n%)	–	18 (39%)	11 (44%)	7 (33%)	0.55
Anti-hypertensives (n%)	–	37 (80%)	18 (72%)	19 (91%)	0.15
Anticoagulation (n%)	–	9 (20%)	5 (20%)	4 (19%)	1

We report mean ± SD, median and interquartile range (IQR) or counts and percent of subsets according to distribution of variables. *Kt*/*V* was calculated according to Daugirdas = − ln((Post BUN/Pre BUN) − (0.008 × Dialysis duration)) + (4 − 3.5 × (Post BUN/Pre BUN)) × (UF/Weight). ANOVA, Mann–Whitney-U-test or cross tables and Chi-Square were used for statistical comparisons of subgroups. *P* < 0.05 was considered statistically significant.

Significant values are in [bold].

BMI = body mass index; Charlson Comorbidity Index adjusted by Liu et al.^40^; COPD = chronic obstructive pulmonary disease; Immunosup. = Immunosuppression; MI = myocardial infarction.

**Figure 1:**
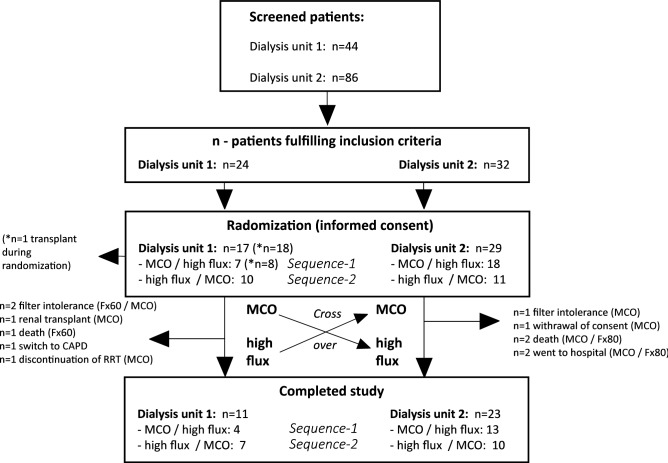
The flow chart shows patient recruitment and inclusion in two participating dialysis units [1: Klinikum rechts der Isar; 2: MVZ KfH Dachau]. Reasons for dropouts are displayed next to horizontal arrows (→). After all, a total of 34 patients completed the whole cross over phase.

Reasons for dropouts and a brief overview of patient recruitment and randomization are displayed in Fig. 1. Two patients (n = 1 Fx60/80, n = 2 MCO) were hospitalized and therefore violated the filter schedule. Adverse events were filter-intolerance (n = 1 Fx60/80, n = 2 MCO). None were severe or needed medical intervention beyond switching to a polysulfone free membrane. Further, three patients died (MCO: n = 1: suicide; Fx60/80: n = 2: sudden cardiac death in known coronary artery disease patients). Other reasons for dropouts included retraction of consent to participate, switch to peritoneal dialysis, successful omission attempt and renal transplant (Fig. 1).

### Primary endpoint analysis

The prespecified primary endpoint was reduction of the inflammatory mediator serum MCP-1 (ITT population), in pre-dialysis (0 h) serum samples after 90 days of MCO versus high flux treatment. The results were essentially the same between ITT- and PP- population.

**Figure 2:**
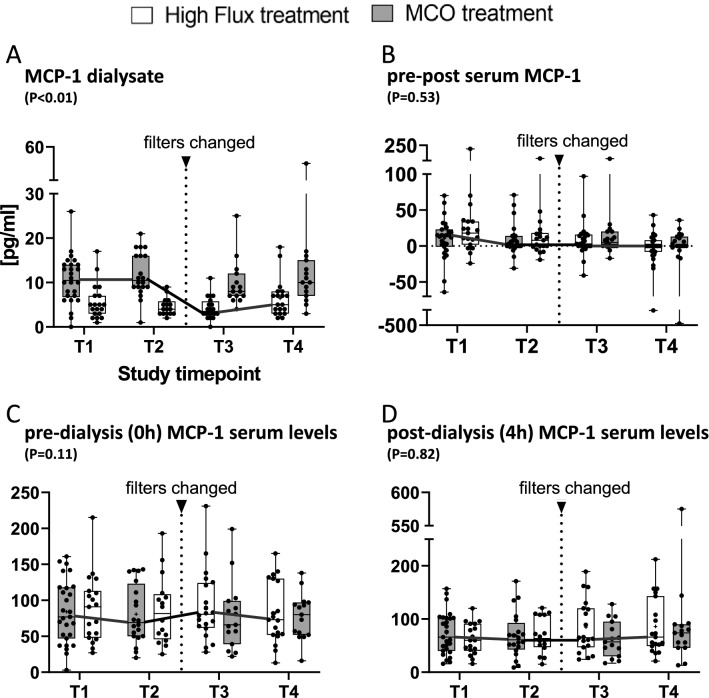
(**A**) Reports mean, and 95%-CIs of dialysate MCP-1 concentrations stratified by treatment (MCO = 1) for all 4 time-points (T1 = Baseline; T2 = after 90 days of treatment with MCO or high flux; T3 = after another 30 days of wash out; T4 = after another days of treatment of high flux or MCO, respectively). (**B**) Δ-values of serum MCP-1 were calculated as: “pre-(0 h)-serum level” minus “post-(4 h)-serum level”. (**C**, **D**) Pre- and post- hemodialysis MCP-1 serum levels stratified by treatment (MCO = 1) are displayed. Herein, Pre-HD MCP-1 is equivalent to long-term (3 months) effects of MCO versus standard high flux membranes. (**A**–**D**) Statistical comparison was done using a linear mixed effect model with subject ID (nested in sequence) as random effect. Treatment (MCO = 1), period and sequence were tested as main effects. A *P* value of < 0.05 was considered statistically significant.

Dialysate-MCP-1 concentrations were significantly higher during MCO versus high flux treatment (10.5 ± 5.9 vs. 5.1 ± 3.8 ng/ml, *P* < 0.001; Fig. 2A; Table 2). Both membranes lead to a significant reduction in pre- (MCO: 81.6 ± 45.0; high flux: 86.4 ± 43.8 pg/ml) versus post- (MCO: 74.1 ± 42.1; high flux: 69.5 ± 37.8 pg/ml) dialysis-MCP-1 serum levels (Table 2). Yet, direct comparisons of Δ pre-post MCP-1 serum levels or MCP-1-AUC (not shown) did not reveal significant differences between MCO- vs. high flux-treatment (Fig. 2B). In line with this, treatment (MCO = 1) was not significantly associated with either pre (0 h)- or post (4 h) dialysis MCP-1 serum levels in LMM analysis (Fig. 2C,D, Table 2). Taken together, although MCP-1 is filtered to a significantly higher extent by MCO membranes, this does not allow for increased serum clearance in a single session. In line with that, there is no significant reduction of MCP-1 pre-dialysis serum levels after a 90-day period (long term reduction; Fig. 2D, Table 3 LMM 0 h model). Taken together the primary endpoint was not reached.

**Table 2:** Primary endpoint analysis.

MCP-1 [pg/ml]	Mean ± SD—high flux	Mean ± SD—MCO	Estimate (95% CI)	*P* value
Dialysate	5.1 ± 3.8	10.5 ± 5.9	6.6 [4.9; 8.3]	< 0.001
Pre-dialysis (0 h)	86.4 ± 43.8	81.6 ± 45.0	− 5.8 [− 12.8; 1.4]	0.11
Post-dialysis (4 h)	74.1 ± 42.1	69.5 ± 37.8	− 1.7 [− 16.0; 12.6]	0.82

The impact of treatment (MCO = 0) on the primary endpoint MCP-1 serum levels was estimated using a linear mixed effects model, with subject ID, nested in sequence as a random effect and treatment (MCO = 1), period and sequence as the main effects. No significant carry over effect was seen for the reported variables. Mean and standard deviation (SD) values for MCP-1 dialysate, pre- or post-dialysis- serum levels were calculated from T1 and T3 stratified by treatment (ITT population). For LMM and paired t-tests a *P* value of < 0.05 was considered statistically significant.

**Table 3:** Extended endpoint analysis—single session and long-term comparison of MCO versus high flux.

	High flux (mean ± SD)			MCO			LMM-4 h		LMM-0 h	
	0 h	4 h	*P*	0 h	4 h	*P*	Est.	*P*	Est.	*P*
MCP-1	86.4 ± 43.8	74.1 ± 42.1	0.001	81.6 ± 45.0	69.5 ± 37.8	0.009	− 1.7	0.82	5.8	0.11
YKL-40	593 ± 419	564 ± 422	0.25	563 ± 376	360 ± 334	< 0.001	− 223	< 0.001	3.9	0.89
IL-8	11.4 ± 6.1	10.6 ± 6.0	0.16	11.3 ± 6.0	10.7 ± 10.0	0.69	− 1.1	0.10	3.94	0.54
IP-10	57.4 ± 47.1	97.9 ± 92.3	0.001	58.1 ± 62.2	91.6 ± 73.8	< 0.001	− 16.6	0.009	1.0	0.84
TNFα	55.8 ± 201.6	588 ± 2629	0.22	28.7 ± 133.8	99.6 ± 491.3	0.21	606	0.45	46.2	0.35
IL-6	7.8 ± 15.5	8.1 ± 7.6	0.86	7.1 ± 6.3	8.2 ± 7.2	0.16	0.5	0.56	− 1.9	0.25
IL-12	0.2 ± 0.5	0.3 ± 0.7	0.28	0.2 ± 0.5	0.3 ± 0.7	0.03	− 0.02	0.60	0.04	0.098
Eotaxin	41.1 ± 24.0	45.5 ± 29.9	0.22	37.7 ± 18.5	39.9 ± 19.1	0.24	4.44	0.02	0.8	0.63
RANTES	1339 ± 726	2454 ± 1391	< 0.001	1224 ± 722	2065 ± 1266	< 0.001	− 75	0.68	− 124	0.22
IL-10	303 ± 628	3703 ± 20,128	0.31	214 ± 593	402 ± 808	0.002	5242	0.39	− 17	0.73
MIG	1029 ± 2027	853 ± 2184	0.04	1002 ± 3037	418 ± 1304	0.04	− 399	< 0.001	119	0.45
IL-4	6.0 ± 21.4	17.2 ± 89.0	0.34	2.9 ± 12.2	10.1 ± 61.2	0.35	− 7.3	0.11	2.1	0.45
IL-13	0.3 ± 0.8	0.4 ± 1.5	0.44	0.3 ± 0.8	0.4 ± 1.4	0.61	− 0.1	0.30	0.06	0.37
IL-1β	50.9 ± 220	70.8 ± 220	0.13	23.7 ± 63.0	31.0 ± 75.3	0.59	− 56	0.13	45.4	0.2
Kt/V *	1.66 ± 0.54		–	1.53 ± 0.31		–	− 0.03	0.62	–	

The table reports mean ± standard deviation (SD) of inflammatory mediators as specified in the left column during MCO versus high flux treatment at T1 and T3—intention to treat population (n = 42 and n = 37 patient × treatment pairs respectively). Interleukin concentrations were assayed as follows: TNFα, IL-10, IL-4, IL-13, IL-1β: fg/ml; MCP-1, IL-8, IP-10, IL-6, Il-12, Eotaxin, RANTES, MIG, IL-4: pg/ml; YKL-40: pg/ml. Pre-versus post-dialysis intra-individual decline or increase was assessed using paired-t-tests within the treatment arms and 0 h and 4 h serum mediator levels, respectively. In the right two columns results from linear mixed effects models (LMM) [using subject ID, nested in sequence as a random effect and treatment (MCO = 1), period and sequence as the main effects] are displayed for post-HD (4 h) values and pre-HD (0 h) values, respectively. Herein, the 4 h LMM compares single session kinetics, whereas the 0 h model tests for long term differences (over a period of 3 months).

Estimate (Est.); Eosinophil chemotactic protein (EOTAXIN), *Kt/*V* was calculated according to Daugirdas = − ln((Post BUN/Pre BUN) − (0.008 × Dialysis duration)) + (4 − 3.5 × (Post BUN/Pre BUN)) × (UF/Weight). Data for calculation of means was used at T1 and T3—intention to treat population (n = 31 and n = 22 patient × treatment pairs respectively); Interleukin (IL); Interferon-inducible protein 10 (IP-10); Monocyte Chemoattractant Protein-1 (MCP-1); Regulated on Activation, Normal T Expressed and Secreted (RANTES, a.k.a. CCL5); Tumor Necrosis Factor alpha (TNFα).

### Secondary endpoints

#### Extended serum mediator profile

**Figure 3:**
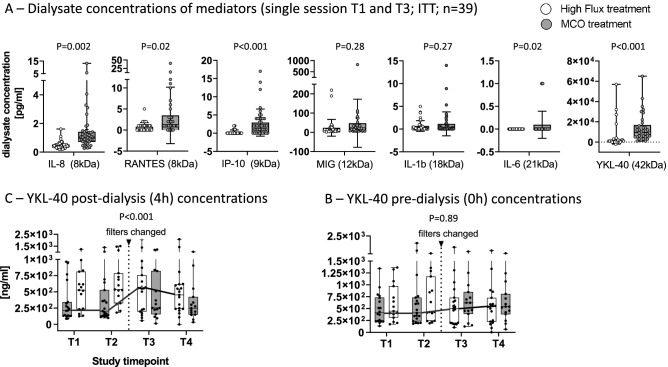
Reports means and 95% confidence intervals. (**A**) Reports mean dialysate concentrations of inflammatory mediators at T1 (baseline) and T3 (after switching treatments) in the intention to treat population. Comparison of treatments: MCO versus high flux dialysis was done using unpaired ANOVA on n = 42 MCO treated versus 39 high flux treated patients × sample pairs, comparable results were obtained on the per protocol population using linear mixed models (not shown). (**B**) Mean YKL-40 serum levels were assayed at all 4 study timepoints in the ITT population after the dialysis session (4 h—sample). (**C**) Mean YKL-40 serum levels were assayed at all 4 study timepoints in the ITT population before the dialysis session (0 h—sample) (**B**, **C)** The thin connecting horizontal lines between timepoints (T1–T4) represent repeated measures from subject IDs within sequence 1 (MCO → wash out → high flux). A linear mixed effects model was built with subject ID, nested in sequence, as a random effect and treatment (MCO = 1), period and sequence as the main effects. A *P* value for treatment < 0.05 in the absence of carry over effect was considered statistically significant.

In addition, we assayed dialysate-concentrations of inflammatory mediators in the ITT population (T1 and T3 data, n = 42 and n = 39 for MCO and high flux, respectively) during MCO versus high flux treatment. Increased dialysate concentrations during MCO treatment were found for IL-8 (MCO vs. high flux: 1.6 ± 2.1 vs. 0.5 ± 0.3 pg/ml, *P* = 0.002), RANTES/CCL-5 (3.5 ± 6.7 vs. 0.8 ± 1.0 pg/ml, *P* = 0.02), IP-10 (2.9 ± 3.7 vs. 0.3 ± 0.6 pg/ml, *P* < 0.001) and IL-6 (0.08 ± 0.2 vs. 0.0 ± 0.0 pg/ml, *P* = 0.02). Other medium sized mediators, i.e., MIG (46.8 ± 124.9 vs. 23.8 ± 43.5 pg/ml, *P* = 0.28) and IL-1β (1.2 ± 2.6 vs. 0.6 ± 1.2 pg/ml, *P* = 0.27) showed the same trend but were not significantly altered in dialysates. Still, the largest mediator tested, namely YKL-40 (approximately 42 kDa), was significantly increased in dialysates (MCO vs. high flux: 15.2 ± 13.6 vs 3.6 ± 11.3 ng/ml, *P* < 0.001) after MCO treatment (Fig. 3A).

Nevertheless, this was accompanied by a significant intra-individual single session mediator reduction during MCO HD only for MCP-1 (0 h vs. 4 h: 81.6 ± 45.0 vs. 69.5 ± 37.8 pg/ml, *P* = 0.009), MIG (0 h vs. 4 h: 1002 ± 3037 vs. 418 ± 1304 fg/ml, *P* = 0.009) and YKL-40 (0 h vs. 4 h: 563 ± 376 vs. 360 ± 334 ng/ml, *P* < 0.001). Increasing concentrations occurred pre versus post MCO HD for IP10 (0 h vs. 4 h: 58.1 ± 62.2 vs. 91.6 ± 73.8 pg/ml, *P* < 0.001), RANTES (0 h vs. 4 h: 1224 ± 722 vs. 2065 ± 1266 pg/ml, *P* < 0.001) and IL-10 (0 h vs. 4 h: 214 ± 593 vs. 402 ± 808 pg/ml, *P* = 0.002). Similar single session kinetics were obtained for high flux membranes (Table 3) with regards to these mediators, with the difference that MCO membranes eliminated significantly more YKL40 in a single session (post session MCO vs. high flux: 360 ± 334 vs. 564 ± 422 ng/ml, *P* < 0.001 Table 3; Fig. 3B). Still, this was not associated with a relevant long term treatment effect [LMM estimate: 4 ng/ml, *P* = 0.89] of MCO membranes (Table 3, Fig. 3C). Thus, whilst MCO-HD eliminates increased small and medium-sized-inflammatory-mediators, it cannot deliver sustained reduction.

#### Secondary endpoint analysis—long term impact on peripheral cellular activation status

**Figure 4:**
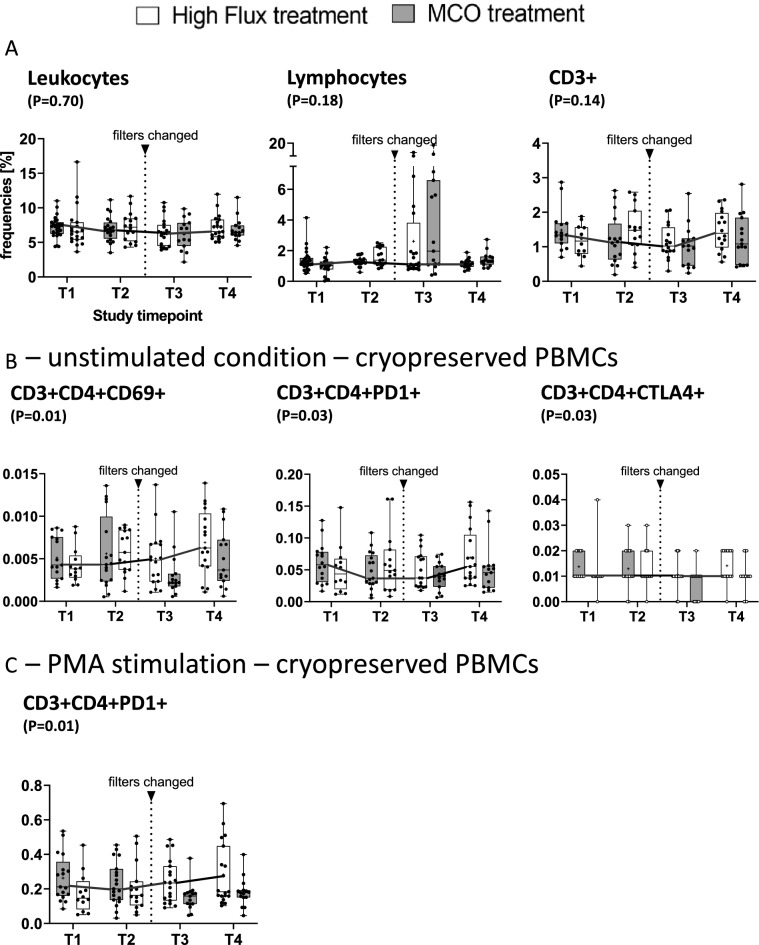
(**A**–**C**) Report mean and 95% CI intervals for leukocytes, lymphocytes and CD3+ cells as reported above stratified by timepoint and treatment (MCO vs. high flux dialysis). *P* values were obtained from linear mixed effect models with subject ID (nested in sequence) with treatment (MCO = 1), period and sequence as main effects. A *P* values of < 0.05 was considered statistically significant. For CD3+ cells as well as (**B**) and (**C**) flow cytometric analysis was performed on re-thawed cryopreserved PBMCs which had been collected before the dialysis session (0 h) at all four study visits. Cells were then either left untreated (**B**) or stimulated with PMA + Ionomycin (**C**)—viability was generally > 85%. Means and SD can be obtained from Table 4. For details with regards to the gating strategy see supplement.

Pre-dialysis peripheral blood counts of leucocytes and lymphocytes and CD3+ T-cells were not differently affected by either MCO or high flux treatment (Fig. 4A and Table 4).

**Table 4:** Secondary endpoint analysis T cell composition and activation.

Cells [G/l]	Mean ± SD—high flux	Mean ± SD—MCO	LMM—estimate	LMM—*P*
Leukocytes	7.21 ± 1.9	6.9 ± 1.8	0.077	0.70
Lymphocytes	1.36 ± 0.5	1.3 ± 0.4	− 0.44	0.18
**Ex vivo unstimulated cells**				
CD3+	1.5 ± 0.6	1.2 ± 0.7	0.10	0.14
CD3+CD4+CD69+	0.006 ± 0.003	0.005 ± 0.004	0.0009	0.012
CD3+CD4+PD-1+	0.07 ± 0.04	0.05 ± 0.03	0.009	0.025
CD3+CD4+CTLA4+	0.01 ± 0.007	0.01 ± 0.006	0.002	0.067
CD3+CD8+CD69+	0.005 ± 0.003	0.004 ± .003	0.0009	0.14
CD3+CD8+PD-1+	0.02 ± 0.02	0.005 ± 0.01	0.003	0.056
CD3+CD8+CTLA4+	0.008 ± 0.007	0.007 ± 0.005	0.001	0.054
**Stimulated cells (LPS)**				
CD3+CD4+CD69+	0.005 ± 0.005	0.005 ± 0.004	0.0007	0.10
CD3+CD4+PD-1+	0.10 ± 0.07	0.08 ± .06	0.01	0.059
CD3+CD4+CTLA-4+	0.012 ± 0.006	0.013 ± .009	− 0.0002	0.79
**Stimulated cells (PMA)**				
CD3+CD4+CD69+	0.8 ± 0.4	0.7 ± 0.4	0.07	0.064
CD3+CD4+PD-1+	0.3 ± 0.2	0.2 ± 0.1	0.03	0.028
CD3+CD4+CTLA-4+	0.013 ± 0.008	0.012 ± 0.006	0.002	0.057

The impact of treatment on ex vivo cell activation (cryopreserved cells prior to HD session start) was modeled in a linear mixed effects model, with subject ID (nested in sequence) as a random effect and treatment (MCO = 0), period and sequence as the main effects, no significant carry over effect (= sequence not significant) was seen for the reported variables. “Scaled identity” was selected as covariance-structure. Mean values for each treatment are reported at the end of each period (T2 and T4, for a total of 34 patients).

Next, we aimed to test the hypothesis whether the transient reduction of inflammatory mediators could positively affect the T cellular immunophenotype in a sustained manner. Pre-dialysis (0h) cryopreserved PBMCs were therefore re-thawed and left untreated or stimulated with LPS or PMA + ionomycin for flow cytometric analysis (data available for 32 patients at T1–T4). Under unstimulated conditions, CD3+CD4+CD69+ (MCO vs. high flux: 0.005 ± 0.004 vs. 0.006 ± 0.003 G/l, *P* = 0.012) but not CD3+CD8+CD69+ cells (0.004 ± 0.003 vs. 0.005 ± 0.003, *P* = 0.14) were significantly reduced during MCO versus high flux dialysis treatment (Fig. 4B, Table 4). In line with this, we found reduced frequencies of CD3+CD4+PD1 expressing cells (MCO vs. high flux: 0.05 ± 0.03 vs. 0.07 ± 0.04 G/l, *P* = 0.03). A similar trend did not reach statistical significance for CD3+CD4+CTLA4+ which appeared to be affected from carry over effect (p(sequence) = 0.03, Fig. 4B). Following stimulation with PMA, the induction of CD3+CD4+PD-1+ cells was significantly reduced after 3 months of MCO treatment (MCO vs. high flux: 0.2 ± 0.1 vs. 0.3 ± 0.2 G/l, *P* = 0.03; Table 4; Fig. 4C). Taken together, MCO treatment was associated with reduced frequencies of CD69+CD3+CD4+ T-cells, as well as reduced PD-1+ CD4 cells following PMA stimulation indicating impact on T cell activation status.

## Discussion

This study was designed to explore the impact of maintenance MCO HD on a broad spectrum of inflammatory mediators and the ex vivo T-cellular immune phenotype of patients. Due to the facts, that MCO membranes are only approved for usage in non-convective HD mode to limit albumin loss, and since HD remains the most widely used dialysis modality the latter was chosen over hemodiafiltration as the comparator^27,28^.

Although the primary endpoint “long term (3 months) reduction of MCP-1 serum levels” was missed, we found a sustained reduction of activated CD4+ T-cells after MCO treatment. In addition, our data demonstrate an elimination equilibrium for most studied mediators during MCO dialysis—specifically that means, that increased mediator clearances (increased dialysate concentrations) could not condition improved short-(single session) and long-term (3 months) elimination-kinetics for most mediators.

Other have independently reported superior and sustained (1–6 months) reduction of larger-sized middle molecules including beta-2-microglobuline (12 kDa) and λ light chain (45 kDa) during MCO versus high-flux-HD or even HDF treatment^20,24,29,30^. Yet, in terms of elimination of inflammatory mediators, they reached conflicting conclusions. On the one hand, Zickler et al. reported “reduced inflammation in chronic dialysis patients” based on reduced leucocyte TNF and IL-6 mRNA expression in their 4-week cross over study, which was lost in an extension phase^26^. On the other hand, Weiner et al. found increased single session and long-term reduction ratios of TNFα but not IL-6 in a 24-week randomized trial^20^. In line with this, a recent systematic review including 3 studies^20,23,26^ could not find a significant long-term reduction of serum IL-6 during MCO sessions^25^. Lastly, Sevinc et al. in their 3 months cross over trial (MCO vs. high-flux HD) in 53 patients report significant reduction of β-2 microglobulin, VEGF and other middle molecules but find similar IL-6, IL-10, IL-17 and Interferon-γ serum levels. They conclude that MCO membranes are not superior to high flux HD in eliminating inflammatory mediators^30^.

Our data complementarily contribute to this debate. Although increased MCP-1 concentrations were detected in dialysate during MCO treatment, the single session serum-kinetics were similar in MCO versus high flux dialysis. We further show that most mediators (8–42 kDa)^31,32^ tested from dialysate samples were filtered to a greater extent during MCO versus high-flux-HD. Still, and in line with most previous data^20,26,29^ we did not find superior single session elimination kinetics of MCO membranes for any mediator except YKL-40. This could be related to either the size of YKL-40 (42 kDa) or a somewhat different biologic kinetic^10^ as discussed below.

Still, why could none of these mediators be reduced sustainably, i.e., by the next HD session? We demonstrate that net elimination (dialysate concentrations) was higher during MCO for most mediators except for IL-1β, and MIG. Therefore, it is tempting to speculate the difference is made up by higher endogenous production or compartment redistribution during dialysis sessions for those mediators studied. Supporting this reasoning, we and others also found a single session increase of certain mediators in pre- versus post-session values, i.e., for IP-10 and RANTES independently of treatment status^33^. In addition, YKL40 has been shown to be induced following LPS challenge with a delay of several hours compared with classical acute-phase-reactants like IL-6^31^. This offers a potential explanation why the significant single session effect on YKL-40 serum levels could not be sustained during MCO HD.

Nevertheless, treatment was associated with a sustained reduction of activated CD69+CD3+CD4+ and PD-1+CD3+CD4+ T cells. Expansion of both cell types has already been described for HD patients and other chronic inflammatory conditions such as type II diabetes^34–36^. Still expansion of activated T-cells in chronic inflammatory condition does not imply immune-functionality but is rather associated with T cell exhaustion and senescence which give way to severe infection and premature aging^19,37,38^. Thus, reducing CD3+CD4+ T cell activation and co-inhibitory receptor/exhaustion marker expression could be a beneficial effect of MCO membranes, which however requires confirmation and further study. Interestingly, Cozzolino et al. in a 2 × 3 months randomized cross over trial in 20 patients observed reduced number of infections during MCO-HD versus high flux HD indicating potential in vivo benefits of MCO membranes^39^. In additional support to our data, Zickler et al. had reported reduced leukocyte mRNA expression of IL-6 and TNFα following four weeks of MCO treatment indicating, that the cellular activation status might in fact be beneficially impacted by MCO although this is not reflected by serum chemistry^26^.

Our study has some strengths and limitations. Strengths include detailed analysis of mediator kinetics including dialysate levels and cellular immune phenotyping. The prospective randomized cross-over design which reached the predefined power cut-off of 34 patients provides low risk for bias with respect to the predefined end point MCP-1 serum kinetics. Nevertheless, with regards to secondary end-point analysis the study should be regarded as exploratory due to multiple testing and a small sample size. In addition, blood and dialysate flow rates were lower than usual likely due to a comorbid cohort (CCI as adjusted by Liu et al. of around nine), which could affect inflammatory mediator clearance despite target dialysis dose (Kt/V). In addition, the endpoint choice does not allow for conclusions with respect to clinical significance of our findings.

Regardless, our data indicate that although MCO membranes do not sustainably reduce serum inflammatory mediators after 3 months of treatment, they are associated with a long-term reduction in peripheral T-cellular hyperactivation. Since these findings were predefined but secondary endpoints, additional studies are needed to determine clinical relevance.

## Methods

### Study design and participants

**Figure 5:**
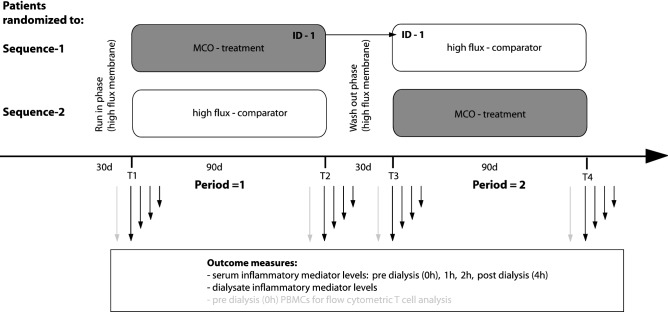
Cross over design, timepoints and sample acquisition.

The study was designed as a multicenter (2 units), interventional, open label, randomized, 2-phase, cross over study. A run in of 30 days was done using the comparator membrane (high flux polysulfone membrane: Fx80 or Fx60 Cor Diax, Fresenius Medical Care). After 3 months (90 days) of treatment with either MCO (Theranova 400, Baxter) or standard high flux membranes (comparator), a wash out of 30 days of high flux treatment was included (see Fig. 5). Outpatients were recruited from 2 HD units: Klinikum rechts der Isar of the Technical University Munich, Bavaria (MRI; unit 1) and Kuratorium für Dialyse und Nierentransplantation (KFH) Dachau, Bavaria (unit 2). Inclusion criteria were age ≥ 18 years, thrice-weekly dialysis sessions with a duration of ≥ 4 h for at least 3 months and written and informed consent. Exclusion criteria were active infection or malignant disease, surgical procedures within 2 weeks prior to inclusion, pregnancy and severe psychologic disorders.

This study was approved by the local ethics committee of the Klinikum rechts der Isar, Technical University Munich (File reference: 559/16S). The trial was conducted following the Declaration of Helsinki, adhering to Good Clinical Practice guidelines. The trial was registered on clinicaltrials.gov prior to its start (“Modulation of Inflammation by Medium Cut-Off Membranes (MCO IF)”: NCT03270371, registered 01.09.2017).

### Study intervention and randomization

After 1 month of “run in”, patients from both units were randomly assigned to receive either 3 months of “90 days MCO membrane treatment → wash out (high flux) → 90 days of high flux”, or vice versa (Fig. 5). 90 days of MCO membrane treatment was defined the intervention. Randomization was done by manual drawing of lots by the study committee group.

### Endpoints and power calculation

The primary endpoint was defined as long term reduction (90 days) of inflammatory mediators, i.e., MCP-1, in pre-dialysis serum samples after 90 days of MCO versus high flux treatment within a 240-day AB/BA cross over design including 30 days run-in (Fx60/80) and wash out (Fx60/80) respectively. Thus 90-day reduction of pre-dialysis = 0 h serum inflammatory mediator concentration were regarded as “long term” sustained reduction.

Secondary endpoints were single-session-kinetics of serum cytokines, including interleukins such as (IL)-6, Tumor necrosis factor alpha (TNFα), chemokines like MCP-1. Dialysate levels of these mediators were assayed (see below). In addition, ex vivo T-cellular activation and stimulation response after 90 days of intervention (MCO) versus comparator (high flux) treatment were prespecified secondary endpoints. Power calculation was done using the online sample size calculator for cross-over studies (Massachusetts General Hospital Mallinckrodt General Clinical Research Center, http://hedwig.mgh.harvard.edu/sample_size/js/js_crossover_quant.html). The following parameters were used: the expected standard deviation for MCP-1 was 150 pg/ml, the expected difference of mean values prior to post intervention was 106 pg/ml (based on PERCI NCT02084381). Herein, 34 patients would be needed to reach a power of 80%.

### Clinical data assessment

Participants' demographics, such as age, gender, comorbidities and medication were assessed using medical records and patient interviews at inclusion and thereafter. Comorbidities were recorded following Liu et al.'s adapted version of the Charlson Comorbidity Index (CCI) as previously described^25,26^. Information about dialysis regimes, vascular access, anticoagulation, effective dialysis time, ultrafiltration rates, Kt/V (Daugirdas) etc. were noted at time of serum sampling and the specified timepoints by study staff and dialysis nurses.

### Sample acquisition and laboratory analysis

Serum samples were collected at 4 different timepoints (T1–T4) pre- dialysis (= 0 h) and 1, 2, or 4 h after mid-week-session-start from vascular accesses. T1 and T2 correspond to the first treatment phase, while T3 and T4 correspond to second treatment phase (see Figs. 1, 5). After 30 min of resting at room temperature serum-samples were centrifuged, aliquoted and frozen at − 80 °C for later analysis. Dialysate samples were collected at all 4 timepoints. For this, a collection system (Supplementary Fig. 1) was connected to the HD machine’s (Fresenius 5008) dialysate outflow line and attached to a peristaltic roller pump running at 10 ml/min. For an average dialysis session of 4 h this resulted in approximately 2.4 L of dialysate, which was mixed at the end of the session to aliquot the final dialysate samples. Spike recovery assays resulted in average recovery of 50–60% per analyte. Routine clinical parameters (e.g., serum sodium, urea, etc.) were analyzed by ISO-accredited laboratories. Serum inflammatory cytokine levels of IL-6, IL-12p70, IL13, Eotaxin, human “interferon-inducible protein 10” (IP-10), MCP-1, “Monokine induced by Gamma-Interferon” (MIG) and “Regulated And Normal T cell Expressed and Secreted” (CCL5, RANTES) were assessed using the BD Flex-sets (BD, Heidelberg, Germany) on a FACS Canto II and BD Diva software following the manufacturer’s instructions. For RANTES the standard concentration had to be doubled to obtain measured values within the range of the standard curve. IL-1β, IL-4, IL-8, IL-10, IL-17, TNFα were assayed using the Enhanced sensitivity Flex sets (BD, Heidelberg, Germany) following the manufacturer’s instructions. IFNγ was not detectable.

YKL-40 protein was measured in duplicates using the commercial Human Chitinase 3-like 1 ELISA Kit (R&D Systems, Inc. Minneapolis, MN, USA) as previously described^10^. Peripheral blood mononuclear cells (PBMCs) were isolated using BD Vacutainer^®^ CPT tubes. Gradient centrifugation and cryopreservation were done within 2 h post sample collection following the manufacturer's protocol. After re-thawing, cells (1 × 10E6/well) were cultured in 96-well plates and were left unstimulated or stimulated with LPS (10 ng/ml) or PMA + Ionomycine (1ug/ml), respectively (8 h, 37 °C). PBMCs were then stained and analysed at once using the antibodies anti-human- CD3, CD4, CD8 to define T cell subsets and anti-human- CD28, CD25, CD69, PD1, and CTLA4 to test for activation (Supplementary Table 1). All surface markers were stained for 30 min at 4 °C. FMOs were used to define the gating of CD69 and CTLA4, respectively (Gating strategies can be retrieved from Supplementary Fig. 2).

### Statistical analysis

SPSS Statistics 23 (IBM, Armonk, NY, USA) was used for statistical analysis. Demographics and patients’ characteristics are reported as mean ± standard deviation (SD), median and interquartile range (IQR), or percent of total, as appropriate. ANOVA, Wilcoxon–Mann–Whitney, or Kruskal–Wallis were used to compare baseline data. For analysis of the prespecified endpoints linear mixed models were built and sequence, period and treatment (MCO = 1) were entered as main effects. Subjects nested in sequence were selected as random intercepts. The primary analysis was performed on the ITT population (PPP was analyzed as a sensitivity analysis). Missing data were not imputed. All tests were performed two-sided and a *P* value of < 0.05 was considered significant.

## Supplementary Information

Supplementary Information.

## Data Availability

Data and analyses presented here are available from the Corresponding Author upon reasonable request.
