# Supplementary material for: A randomized prospective cross over study on the effects of medium cut-off membranes on T cellular and serologic immune phenotypes in hemodialysis
Source: Sci Rep. 2022 Sep 30;12:16419. doi: 10.1038/s41598-022-20818-z (PMC9524345; doi:10.1038/s41598-022-20818-z)
Supplement: Supplementary file 1 — Supplementary Information. [file 41598_2022_20818_MOESM1_ESM.docx]

**Supplementary material: A prospective cross over study on the effects of medium cut-off membranes on T cellular and serologic immune phenotypes in hemodialysis.**

Georg Lorenz^1^ *#, Yuli Shen^1^ ^6^*, Renate Hausinger^1^, Caroline Scheid^1^, Marie Eckermann^1^, Sophia Hornung^1^, Joana Cardoso^1^, Maciej Lech^1,2^, Andrea Ribeiro^1,2^, Bernhard Haller^3^, Christopher Holzmann-Littig^1^, Dominik Steubl^1^, Matthias C. Braunisch^1^, Roman Günthner^1^, Andreas Poschenrieder^4^, Britt Freitag^5^, Mario Weber^5^, Peter Luppa^4^, Uwe Heeman^1^, Christoph Schmaderer^1^

^1^ Technical University of Munich, School of Medicine, Klinikum rechts der Isar, Department of Nephrology, Germany

^2^ LMU Klinikum Department of nephrology Ludwig-Maximilian University, Munich, Germany

^3^ Institute for AI and Informatics in Medicine, Klinikum rechts der Isar, Technical University Munich, Germany

^4^ Department of clinical chemistry, Klinikum rechts der Isar, Technical University Munich, Germany

^5^ [MVZ KfH-Gesundheitszentrum Emmering / Dachau, Dachau Germany](https://www.google.com/url?sa=t&rct=j&q=&esrc=s&source=web&cd=&cad=rja&uact=8&ved=2ahUKEwid5o2c_ZD0AhUt57sIHeJoAbgQFnoECAgQAQ&url=https%3A%2F%2Fwww.kfh.de%2Fmvz%2Femmering-dachau%2Fnephrologie%2F&usg=AOvVaw1weWQ_pP4WFTSKPHiP7kcy)

^6^ Nephrology & Rheumatology Department, Longgang District People’s Hospital of Shenzhen

& The Third Affiliated Hospital (Provisional) of The Chinese University of Hong Kong, Shenzhen, 518172, China

* these authors contributed equally to this work

# corresponding author

**Supplementary Table 1**. Monoclonal antibodies and conjugates

| **Application** | **Target antigen/** **fluorescence** | **Catalog nr.** | **Clone** | **Company** |
| --- | --- | --- | --- | --- |
| Viability dye | Propidium Iodide | P3566 | 1820546 | Thermo Fisher Scientific, Waltham, USA |
| Viability dye | Fixable Viability Dye eFluor 780 | 65.0865.14 | 2039434 | Thermo Fisher Scientific, Waltham, USA |
| T cells surface antigens | Anti- Human CD25 PE-Dazzle | 356126 | M-A251 | Biolegend, San Diego, USA |
|  | Anti- Human CD3 BV605 | 317322 | OKT3 | Biolegend, San Diego, USA |
|  | Anti- Human CD4 BV785 | 300554 | RPA-T4 | Biolegend, San Diego, USA |
|  | Anti- Human CD8 BV510 | 301048 | RPA-T8 | Biolegend, San Diego, USA |
|  | Anti- Human CD69 AlexaFluor700 | 310922 | FN30 | Biolegend, San Diego, USA |
|  | Anti- Human CD28 APC | 302912 | CD28.2 | Biolegend, San Diego, USA |
|  | Anti- Human PD-1 PE | 329906 | EH12.2H7 | Biolegend, San Diego, USA |
|  | Anti- Human CTLA4 PE/Cy7 | 349914 | L3D10 | Biolegend, San Diego, USA |

T cells were stained using antibodies for CD25, CD45RO, CD3, CD4, CD8, CD28, CD69, PD1, and CTLA4+. FMOs were used to define the gating of CD69 and CTLA4, respectively.All surface markers were stained for 30mins at 4°C.

**Supplementary table 2:** Baseline patients’ characteristics for the per-protocol population

| **Parameter** | **Missing data** | **Overall** | **MCO 🡪 Fx60/80** | **Fx60/80 🡪 MCO** | **P -value** |
| --- | --- | --- | --- | --- | --- |
| **Number of patients** | **-** | **34** | **17** | **17** | **-** |
| **Age (years)** | - | 71 [62, 79] | 77 [61, 79] | 67 [52, 78] | 0.36 |
| **Sex, male (n%)** | - | 24 (71%) | 13 (77%) | 11 (65%) | 0.71 |
| **BMI (kg/m^2^)** | - | 26 ± 5 | 28 ± 5 | 24 ± 5 | 0.64 |
| **Upper arm circumference (cm)** | 2 | 29 ± 5 | 30 ± 5 | 29 ± 5 | 0.46 |
| **Cause of CKD** | - |  |  |  | 0.4 |
| **- Diabetes / hypertension** | - | 15 (44%) | 9 (53%) | 6 (35%) | - |
| **- Glomerulonephritis** |  | 5 (15%) | 4 (24%) | 1 (6%) | - |
| **- Systemic disease** |  | 1 (3%) | 0 (0%) | 1 (6%) | - |
| **- hereditary cause** |  | 6 (18%) | 2 (12%) | 4 (24%) | - |
| **- other** |  | 7 (20%) | 2 (12%) | 5 (29%) | - |
| **CCI** | - | 9 [6, 10] | 9 [5, 12] | 9 [6, 10] | 0.79 |
| **Diabetes mellitus (n%)** | - | 11 (32%) | 6 (35%) | 5 (29%) | 1 |
| **History MI (n%)** | - | 8 (24%) | 2 (12%) | 6 (35%) | 0.26 |
| **Coronary heart disease (n%)** | - | 15 (44%) | 9 (53%) | 6 (35%) | 0.49 |
| **Atria Fibrillation (n%)** | - | 13 (38%) | 8 (47%) | 5 (29%) | 0.48 |
| **COPD(n%)** | - | 8 (24%) | 4 (24%) | 4 (24%) | 1 |
| **HD vintage [months]** | - | 36 [29,86] | 46 [32, 88] | 33 [10, 78] | 0.27 |
| **Residual renal function [ml], n=15** | - | 720 [400, 1090] | 720 [375, 1075] | 563 [363, 1155] | 1 |
| **Access (catheter)** | - | 7 (21%) | 0 (0%) | 7 (41%) | **0.007** |
| **Anticoagulation (citrate / agatra n=1)** | - | 5 (15%) | 3 (18%) | 2 (12%) | 0.60 |
| **Effective session duration [h]** | - | 4.2 ± 0.3 | 4.4 ± 0.3 | 4.1 ± 0.2 | 0.59 |
| **Ultrafiltration rate [ml/h]** |  | 525 ± 189 | 535 ± 162 | 514 ± 217 | 0.24 |
| **Blood flow rate [ml/min]** | - | 238 ± 47 | 243 ± 49 | 232 ± 46 | 0.73 |
| **Dialysate flow [ml/min]** | - | 378 ± 133 | 382 ± 145 | 374 ± 125 | 0.99 |
| **Dialysate Na+ [mmol]** | - | 138 [138, 138] | 138 [138, 138] | 138 [138, 138] | 0.79 |
| **Dialysate K+ [mmol]** | - | 3 [2,3] | 2 [2,3] | 3 [2,3] | 0.13 |
| **Dialysate Mg2+[mmol]** | - | 1 [0.75, 1] | 0,75 [0.75, 1] | 1 [0.88, 1] | 0.11 |
| **Dialysate Ca2+ [mmol]** | - | 1,25 [1.25, 1.25] | 1,25 [1.25, 1.25] | 1.25 [1.25, 1.25] | 0.79 |
| **Dialysate HCO3- [mmol]** | - | 32 [32, 32] | 32 [32, 32] | 32 [32, 32] | 0.63 |
| **Dialysis unit 1 (n%)** | - | 11 (32%) | 4 (24%) | 7 (41%) | 0.47 |
| **Immunosup. (n%)** | - | 2 (6%) | 1 (6%) | 1 (6%) | 0.60 |
| **Statin(n%)** | - | 11 (32%) | 6 (35%) | 5 (29%) | 1 |
| **Anti-hypertensives (n%)** | - | 25 (74%) | 10 (59%) | 15 (88%) | 0.12 |
| **Anticoagulation(n%)** | - | 6 (18%) | 3 (18%) | 3 (18%) | 1 |

Abbreviations: BMI = body mass index; COPD = chronic obstructive pulmonary disease; Immunosup. = Immunisuppression; MI = myocardial infarction.


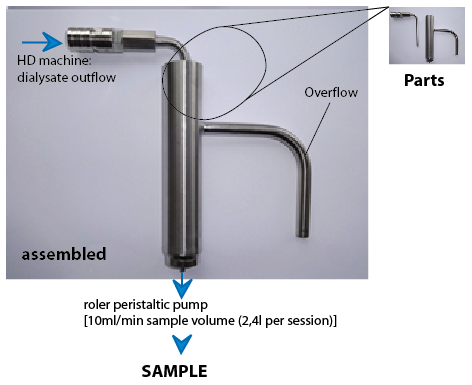


**Supplementary figure 1:** The device used to sample an “average dialysate” specimen **consists of a** collection siphons (see pictures) attached to a continuously working peristaltic roler pump (set at 10ml/min). This way fractions of the dialysates with high (beginning of HD session) or low (end of HD session) mediator levels were collected in equal shares during an average dialysis session.


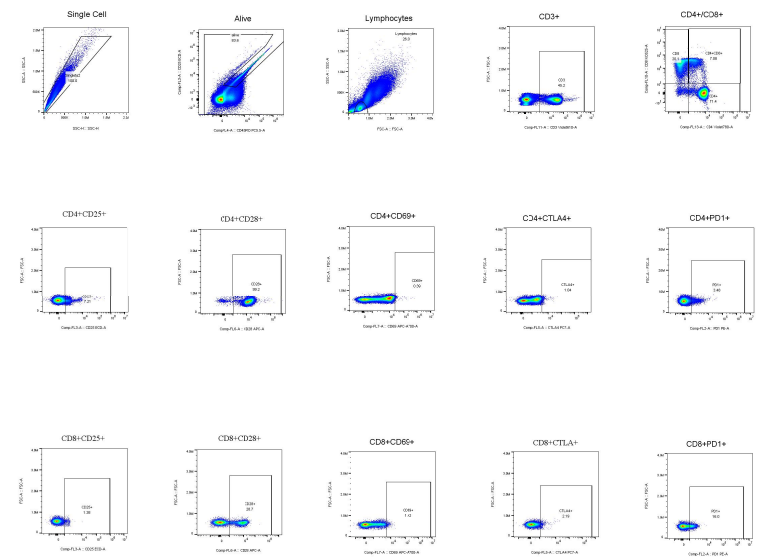


**Supplementary figure 2:** T cell gating strategy for cryopreserved PBMCs.
